# Electrospun Nanofibrous Poly (Lactic Acid)/Titanium Dioxide Nanocomposite Membranes for Cutaneous Scar Minimization

**DOI:** 10.3389/fbioe.2019.00421

**Published:** 2019-12-20

**Authors:** Teresa C. O. Marsi, Ritchelli Ricci, Tatiane V. Toniato, Luana M. R. Vasconcellos, Conceição de Maria Vaz Elias, Andre D. R. Silva, Andre S. A. Furtado, Leila S. S. M. Magalhães, Edson C. Silva-Filho, Fernanda R. Marciano, Andrea Zille, Thomas J. Webster, Anderson O. Lobo

**Affiliations:** ^1^Institute of Research and Development, University of Vale Do Paraiba, São José dos Campos, Brazil; ^2^Department of Bioscience and Oral Diagnosis, Institute of Science and Technology, São Paulo State University, São Paulo, Brazil; ^3^Scientific and Technological Institute, Brasil University, São Paulo, Brazil; ^4^Air Force Academy, Brazilian Air Force, Pirassununga, Brazil; ^5^LIMAV - Interdisciplinary Laboratory for Advanced Materials, Materials Science & Engineering Graduate Program, UFPI-Federal University of Piaui, Teresina, Brazil; ^6^Department of Physics, Federal University of Piaui, Teresina, Brazil; ^7^Department of Textile Engineering, Centre for Textile Science and Technology, University of Minho, Guimarães, Portugal; ^8^Department of Chemical Engineering, Northeastern University, Boston, MA, United States

**Keywords:** PLA, nanocomposites, electrospinning, cutaneous scarring, gene expression, *in vivo*

## Abstract

Poly (lactic acid) (PLA) has been increasingly used in cutaneous tissue engineering due to its low cost, ease of handling, biodegradability, and biocompatibility, as well as its ability to form composites. However, these polymers possess a structure with nanoporous that mimic the cellular environment. In this study, nanocomposites are prepared using PLA and titanium dioxide (TiO_2_) (10 and 35%—w/w) nanoparticles that also function as an active anti-scarring agent. The nanocomposites were prepared using an electrospinning technique. Three different solutions were prepared as follows: PLA, 10% PLA/TiO_2_, and 35% PLA/TiO_2_ (w/w%). Electrospun PLA and PLA/TiO_2_ nanocomposites were characterized morphologically, structurally, and chemically using electron scanning microscopy, transmission electron microscopy, goniometry, and X-ray diffraction. L929 fibroblast cells were used for *in vitro* tests. The cytotoxic effect was evaluated using 3-(4,5-dimethylthiazol-2-yl)-2,5-diphenyltetrazolium bromide assays. Versicam (VCAN), biglicam (BIG), interleukin-6 (IL6), interleukin-10 (IL-10), and type-1 collagen (COL1A1) genes were evaluated by RT-qPCR. *In vivo* tests using Wistar rats were conducted for up to 15 days. Nanofibrous fibers were obtained for all groups that did not contain residual solvents. No cytotoxic effects were observed for up to 168 h. The genes expressed showed the highest values of versican and collagen-1 (*p* < 0.05) for PLA/TiO_2_ nanocomposite scaffolds when compared to the control group (cells). Histological images showed that PLA at 10 and 35% w/w led to a discrete inflammatory infiltration and expression of many newly formed vessels, indicating increased metabolic activity of this tissue. To summarize, this study supported the potential of PLA/TiO_2_ nanocomposites ability to reduce cutaneous scarring in scaffolds.

## Introduction

The standard treatment for skin lesions uses dressings that come in direct contact with the injured region. By replacing these dressings with scaffolds, this treatment becomes non-invasive to minimally invasive along with other positive outcomes such as a reduction in patient recovery times, medical costs and consumption of scarce and valuable health-care resources around the world for treatment of large-scale musculoskeletal injuries with traumatic lesions, birth defects and surgical excisions (Bardosova and Wagner, 2015; Beyth et al., 2015; Walmsley et al., 2015; Ghannadian et al., 2018). Bioabsorbable and bio-degradable polymers have been shown enough mechanical properties that accelerate the cell proliferation process while providing antimicrobial protection. This makes them promising materials for biomedical applications as they have been shown to optimize the tissue repair process which in turn speeds up patient recovery times (Simoes, 2011; Wang et al., 2014; Fonseca et al., 2015). Among the various polymers, poly (lactic acid) (PLA) has proven to be an ideal candidate material due to its mechanical properties, good biocompatibility, low cost, and the adjustable degradation profile with CO_2_, H_2_O (Hidalgo et al., 2013; Tawakkal et al., 2014; Annunziata et al., 2015; Toniatto et al., 2017) and polyester as by-products, either from the esterification of lactic acid and its fermentation (Toniatto et al., 2017).

PLA-based nanofibrous fibers have large surface areas, allowing them to interact with large volumes of other substances in their environment. This is a distinguishing feature of this material (Bayon et al., 2016; Toniatto et al., 2017; Salles et al., 2018). The fiber surfaces have several characteristics that make them similar to the extracellular matrices (ECMs) used in biomedical applications. In addition, the interaction of the cells and the substrate influences their morphology, proliferation, and viability (Braunger et al., 2017). ECMs have been assumed to be inert structures that consist of proteins and polysaccharides that are synthesized and secreted by cells. Their sole purpose was once considered to fill up extracellular space. However, recent research indicates that ECMs perform other key roles. They function as scaffolds, aid in cell binding, allow for tissue formation, and play an important role in the control of cell growth, differentiation, adhesion, migration, proliferation, and angiogenesis (Villarreal-Gómez et al., 2016; Saldin et al., 2017).

Studies have shown that titanium dioxide (TiO_2_) nanoparticles are highly biocompatible and have good physical, chemical, mechanical, and biological properties. These nanoparticles have a variety of uses in many biomedical applications. One study found that they help increase protein absorption and reduce infections caused by both Gram-positive and Gram-negative bacteria (Roy et al., 2007; Liou and Chang, 2012; Kandiah et al., 2014; Wu et al., 2014; Toniatto et al., 2017).

Electrospinning of polymers can be used to generate three-dimensional fibrous structures and is therefore used to produce mats with these polymers. The electrospun mats closely resemble natural ECM (Villarreal-Gómez et al., 2016; Toniatto et al., 2017) and are capable of supporting cell adhesion and proliferation. Due to their inherent material properties, these mats not only provide a three-dimensional structure but are also biocompatible, bioabsorbable, and have antibacterial properties, making them extremely desirable for use in scaffolds and medical devices (Roux et al., 2013; Stocco et al., 2018). PLA/TiO_2_–nanofibrous fibers produced by electrospinning are being studied in order to evaluate their potential in dressing and wound healing applications (Bayon et al., 2016; Toniatto et al., 2017; Ghosal et al., 2018; Salles et al., 2018).

In prior studies, we have shown that PLA/TiO_2_-based scaffolds have bactericidal properties and do not exhibit cytotoxicity (Toniatto et al., 2017). Here, we further evaluated the toxicity of electrospun PLA in fibroblast cells and rats (skin model) and compare the results when the material is embedded two different concentrations of TiO_2_ nanoparticles PLA/TiO_2_−10% w/w (PLA—A) and PLA/TiO_2_−35% w/w (PLA—B). We also investigated their potential to upregulate specific genes related to the regenerative process. These electrospun scaffolds are biocompatible and showed no inflammations in rats. They were also found to have upregulated the versicam and type-1 collagen genes. These results provide a strong rationale to use PLA/TiO_2_ scaffolds as dressings for skin lesion applications.

## Materials and Methods

### Materials

Chloroform, N, N-diethylformamide (DMF), ethyl alcohol, Dulbecco's MEM (DMEM), Fetal Bovine Serum (FBS), 3-4,5-dimethylthiazol-2-yl-2,5-diphenyltetrazolol bromide, neutral buffered Formalin, Hematoxilin and Eosin were purchased from Sigma-Aldrich® (USA). PLA (2003D, with 4.30% of D-lactic acid monomer) was donated by NatureWorks (Minnetonka, Minnesota, United States). TiO_2_ nanoparticles were donated by Evonik Degussa (AEROXIDE® TiO_2_ P25, Essen, North Rhine-Westphalia, Germany). NCTC clone 929 (L CELL, L-929) cells were purchased from a bank cell in Rio de Janeiro, Brazil. 24-wells plates were purchased from Ciencor®. RNAeasyTM mini kit was purchased from Qiagen (São Paulo, Brazil). Versicam, biglicam, interleukins-6, interleukins-10, collagen-1 genes, complementary DNA (cDNA), RNA, and GoTaq® qPCR Master Mix amplifier kit were purchased from Promega (São Paulo, Brazil).

### Electrospinning of PLA/TiO_2_ Nanocomposite Membranes

Three types of solutions were prepared: PLA, PLA with 10% TiO_2_ by weight, and PLA with 35% TiO_2_ by weight. In the first step, 0.09 g of PLA was dissolved in 0.6 mL of chloroform at room temperature for about 150 min in closed system. Three sets of these PLA solution compositions were prepared. Afterwards in two separate containers, TiO_2_ nanoparticles (0.01 and 0.05 g, respectively) were dispersed in 0.4 mL DMF using a tip ultrasound (Sonics, VCX 500) for ~90 min. A third container with 0.4 mL DMF was also prepared without the addition of TiO_2_. Subsequently, each of the three DMF solutions (two of them containing TiO_2_ of different concentrations, and one without TiO_2_) was added to each of the three PLA solutions in chloroform and then stirred magnetically for 20 h in an enclosed system at room temperature. Table 1 summarizes the masses and volumes of three prepared solutions. The electrospinning process was performed under a temperature and humidity-controlled exhaust hood (at a temperature of 25 ± 2°C and relative humidity of 30–40%). The electrospinning parameters/apparatus used were: 12 kV (Bertan 203R), syringe (5 mL, BD®), metal needle (23G, Inbras), infusion rate (0.05 mL/h), on a collector covered with aluminum foil (100 × 100 × 1 mm, at a distance of 10 cm) and total time of 30 min.

**Table 1:** Description of produced solutions prior electrospinning process.

**Scaffolds**	**PLA (g)**	**TiO_**2**_ (g)**	**Chloroform (mL)**	**DMF (mL)**
PLA	0.09	–	0.6	0.4
PLA—A	0.09	0.01	0.6	0.4
PLA—B	0.09	0.05	0.6	0.4

### Characterization of Structural, Physical, and Chemical Properties

The samples were characterized after 24 h under vacuum. Scanning electron microscope (SEM, Zeiss EVO MA10) was used to analyze the morphology and determine the diameters of the fibers. To aid the analysis, a thin layer of gold (~10 nm thickness) was deposited using sputtering under an Argon plasma at a pressure of 0.2 mbar under an applied current of 30 mA for 2 min. The micrographs were obtained using magnifications of 500x, 1,000x, and 5,000x. Images were obtained using the SEM and analyzed using ImageJ software to establish the mean diameters for the fibers, and the mean and standard deviations of the data were calculated. Transmission electron microscopy (TEM, Philips CM120) was used to evaluate the homogeneity of the TiO_2_ nanoparticles incorporated into the fibers. To perform the analysis, the fibers were collected for 5 s onto a copper transmission grid of 3.05 mm in diameter. The grid was positioned at a working distance of 10 cm, and the material was deposited for a few seconds, until a thin layer was formed on the grid.

A goniometer (Krüss DSA 100) operating in dynamic mode was used to measure the angle between the scaffolds and air using water and diiodomethane. Two microliters of deionized water was dropped on each scaffold and images were recorded after 1 min. This test was performed on 5 samples, and the mean and standard deviations for the results were calculated.

TGA measurements were carried out in a STA 7200 Hitachi (Tokyo, Japan). TGA plots were obtained within the range of 25–900°C under nitrogen atmosphere (200 mL·min^−1^) at 10°C·min^−1^. Specimens were left at room temperature (25°C) until equilibrium was reached and placed in an aluminum pan. Data was plotted as weight loss percentage vs. temperature, and the mass of dried residues was calculated for each case. The derivative thermogravimetric (DTG) analysis was also performed to identify the maximum peaks of the thermal transformation events.

DSC analyses were carried out in a Mettler-Toledo DSC822 instrument (Giessen, Germany). Analyses were carried out in an aluminum sample pan under nitrogen atmosphere with a flow rate of 20 mL min^−1^ and heating rate of 10°C min^−1^. In order to eliminate the thermal history of the material, the first heating cycle was obtained in the range of 0–110°C, afterwards it was cooled down to 0°C and heated again up to 500°C. The graph was plotted as heat flow vs. temperature.

The tensile strength, elongation at break and fracture strain of the nanofibers were measured using a texture analyzer (TA.XT plus, Stable Micro Systems Ltd., Vienna, UK). Rectangular samples of the polymeric scaffolds were specifically cut to have dimensions of 10.00 × 30.00 × 0.10 mm and fixed with the probe provided by instrumentation attached to a 5 kg load cell. Measurements were recorded at 25°C with a strain rate of 1 mm.min^−1^ (*N* = 3).

X-ray diffraction (XRD) (PANalytical X'Pert Pro diffractometer) using a monochromatic X-Ray CuKα radiation, was used to study the crystalline structure of the samples. Data were collected over a range of 10–80° using a scanning speed of 0.08 degrees per minute. Data was analyzed using HighScore 3.0a software (PaNalytical, Almelo, Netherlands) for phase identification. The crystalline index was calculated as the ratio of the crystalline scattering fraction to the total crystalline and amorphous scattering.

### Biomedical Characterizations of PLA/TiO_2_ Nanocomposite Membranes

Prior to performing any biological assay, the samples were vacuum dried. For the biological assays, L929 fibroblasts cells were cultured in Dulbecco's Modified Eagle's Medium supplemented with fetal bovine serum (90:10 v/v) and kept in a 5% CO_2_ atmosphere at 37°C for 7 days to obtain a confluence layer. The polymer sheets were sterilized in ultraviolet radiation and then placed in 70% ethyl alcohol, washed with phosphate-buffered saline, and hydrated with the DMEM/FBS medium prior to use in the biological assays (Lobo et al., 2008).

A 3-(4,5-dimethylthiazol-2-yl)-2,5-diphenyltetrazolium bromide (MTT) assay was used to analyze the scaffold (PLA only, PLA—A, and PLA—B) scaffolds for cytotoxicity. Twenty thousand cells were plated in a 24-well plate. After 24 h, a 10 × 10 mm square piece of each of the scaffold types was placed on separate plates. After both 24 and 168 h, 100 μL MTT (1 mg/mL) was added to the culture medium in each well. The plate was then covered with an aluminum foil and incubated for 2 h in an oven with a consistent a 5% CO_2_ atmosphere and 37°C temperature (Lobo et al., 2008). The MTT was then removed and 100 μL of dimethyl sulfoxide (DMSO) was added in each sample. The absorbance was then measured using a spectrophotometer (570 nm wavelength; instrumentation by AsysHitech GmbH, Eugendorf, Austria). Cells were used as negative controls and latex fragments (10 × 10 mm) as positive controls for cytotoxicity tests. To normalize the results, the absorbance of a blank sample and DMSO were also measured. Gene expression analysis by RT-qPCR, and extraction of total RNA from adhered cells on scaffolds were performed after 7, 14, and 21 days. Versicam, Biglicam, interleukins-6, interleukins-10, and collagen-1 (COL1A1) genes were expressed upon performing RT-qPCR. The integrity of the RNA was evaluated using agarose gel electrophoresis (1.5%) and analyzed using the 18S and 28S bands. Thereafter, the outer diameter was measured (at 260 and 280 nm wavelengths using Nano Drop 2000 manufactured by Thermo Fisher) and the concentration and purity of the RNA sample was determined. Values A260/A280 between 1.8 and 2.0 were accepted. For the synthesis of deoxyribonucleic acid (cDNA), 2.0 μg of RNA obtained via reverse transcription was used following the manufacturer's instructions. The cDNA was amplified and an ABI PRISM 7500 sequence detector (Applied Biosystems, USA) was used for quantitative analysis of the gene expression. The primers analyzed are listed in Table 2. The conditions/parameters applied during this analysis were 95°C (for 5 min), 40 cycles of 15 min each at 95°C, 60°C (for 1 min), and a final cycle of 5 min at 72°C. Each experiment was repeated three times and the data was normalized according to the expression of the reference gene using the selection of the most appropriate endogenous control. Three reference genes were used: Glyceraldehyde 3-phosphate dehydrogenase (GAPDH), ribosomal 18S RNA (18SrRNA), and beta-beta smooth muscle (β-actin); β-actin was the preferred reference gene. The ΔΔCt method acquires average cycle limit values (Cts) of the target genes and compares them with the Cts of the average reference gene. Relative gene expression was calculated using the 2^−ΔΔCt^ method (Livak and Schmittgen, 2001).

**Table 2:** Description of the gene used in RT-qPCR.

**Gene**	**Gene name**	**Primer sequences**	**Ref. Fast Pubmed**
VCAN	Versicam	5′-CAAACCCTGCCTCAACGGAGG-3′ 5′-CCTTCAGCAGCATCCCATGTGCGT-3′	NM_001101
BGN	Biglycan	5′-GATGGCCTGAAGCTCAA-3′ 5′- GGTTGTTGAAGAGGCTG-3′	NM_199173
COL1A1	Type I Collagen alpha 1	5′-CCCTGGAAAGAATGGAGATGAT-3′ 5′-ACTGAAACCTCTGTGTCCCTTCA-3′	NM_000088.3
IL6	Interleukin 6	5′-AGCCAGAGCTGTGCAGATGA-3′ 5′-GCAGGCTGGCATTTGTGGTT-3′	NM_031168.2
IL-10	Interleukin 10	5′-AGCCAGCAGCTCTCAAGTC-3′ 5′-GTGTTCAGTGTGGTCCTGGAT-3′	NM_010548.2

The RNA samples were analyzed using the NanoDrop ND-1000 spectrophotometer (Thermo Fisher Scientific, USA) at wavelengths of 260 nm for RNA and 280 nm for the protein.

The primers were designed with the aid of the RTD program (Integrated DNA Technologies, www.idtdna.com) and Primer 3 software (frodo.wi.mit.edu/cgi-bin/primer3/primer3_www.cgi).

### Experimental Model—*in-vivo*

All *in-vivo* procedures were performed in accordance with ethical standards. The testing protocol was approved by the Brazilian committee (10/2015-CEUA/ICT/CJSC-UNESP). Six male Wistar rats (*Rattus norvegicus*) aged 90 days and weighing between 350 and 400 g were used. The animals were provided with food and water *ad libitum*. The PLA, PLA—A, and PLA—B samples were implanted in the rat dorsal subcutaneous tissue (*n* = 2). The apparatus was cleaned using 70% ethanol and sterilized for 2 h using UV radiation and surgically inserted using procedures described in Camargo et al. (2010). The rats were euthanized 15 days after the surgery.

For histological analysis, a 10% neutral buffered formalin was applied on the surgical sites. After 48 h, the specimens were processed using paraffin embedding. The paraffin block was oriented parallel to the long axis of the material, and serial sections of 5 μm thickness were cut. Theses sections were then stained with hematoxylin and eosin. Histological qualitative evaluation was conducted using microscopic analysis.

### Statistical Analysis

A sample size of 5 has been used in this study. The data was analyzed using two-way analysis of variance (ANOVA) followed by a Tukey's test (GraphPad Prism software, v. 5.01). A value of *p* < 0.05 was considered statistically significant.

## Results

### Characterization of the Electrospun Scaffolds

**Figure 1:**
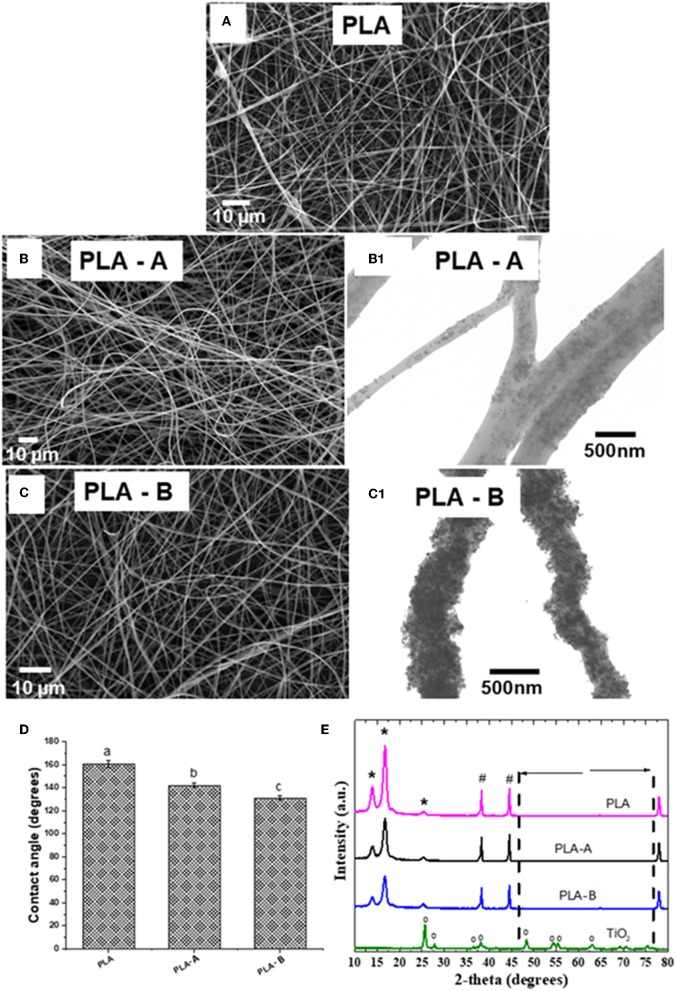
**(A)** Micrograph for PLA fiber without TiO_2_ addition; micrographs of polymer solutions with **(B)** PLA—A; **(C)** PLA—B. **(D)** Contact angles measured. **(E)** XRD of the developed membrane. Images obtained by TEM **(B.1)** PLA—A, **(C.1)** PLA—B. * and ^#^ referred to typical PLA crystalline planes. ° referred to typical TiO_2_ crystaliine planes.

**Figure 2:**
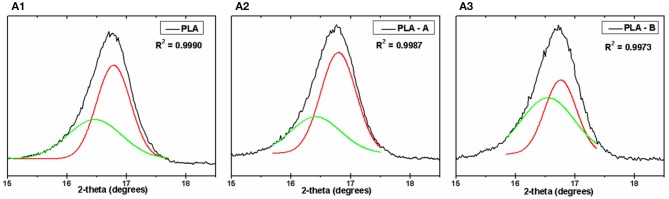
X-ray diffractograms deconvoluted in the intervals presented for: PLA **(A.1)**, PLA—A **(A.2)**, and PLA—B **(A.3)**.

Figures 1A–C shows SEM micrographs of PLA, PLA—A, and PLA—B nanofibrous scaffolds, respectively. The homogeneity of the nanofibers can be observed; the diameters of the nanofibers appear to be similar and they no obvious deformation and free of beads. The mean values of the diameters of the nanofibers in the samples of PLA with 10 and 35% TiO_2_ were 332 ± 108 and 332 ± 95 nm, respectively—slightly larger than that observed in the PLA sample without TiO_2_ (315 ± 87 nm). The images obtained by TEM showed that TiO_2_ nanoparticles were homogeneously dispersed within the PLA fibers at both the concentration levels of 10 and 35%—w/w (Figures 1B.1,C.1). Figure 1D shows contact angle measurement using water. It can be observed that incorporation of TiO_2_ nanoparticles causes an observable decrease in the contact angle. The PLA, PLA—A, and PLA—B samples had contact angles of 160.0 ± 3.0, 140.0 ± 2.1, 130.0 ± 2.2°, respectively. XRD measurements showed that TiO_2_ has a different growth process in the single crystalline phase corresponding to the anatase phase (Figure 1E). The preferred orientation plane is the crystalline plane (101) around of 2θ = 25°, which is typical of the anatase phase and the rutile phase, indicating a high purity of the material. This is confirmed by the peaks, 2θ = 37, 49, 54, 56, 63, 70, and 76° at the corresponding crystallographic planes (1 0 3), (2 0 0), (2 1 1), (2 1 1), (2 0 4), (1 1 6), and (2 1 5) (Dinari and Haghighi, 2017; Pava-Gómez et al., 2018). The peaks at 25, 37, and 49° are characteristic of TiO_2_ and are clearly observed when they are part in the fibers. The intensity of the XRD peaks was lower in the samples that contained the TiO_2_ than in those without TiO_2_ (Figures 2A.1–A.3). The measured crystalline index was 89, 87, and 82% for PLA, PLA—A, and PLA—B samples, respectively.

Figure 2 shows deconvolutions obtained from XRD, as shown in Figure 1E. The amplitude of the peak found between 16.45 and 16.80° was used to determine the proportion of pure PLA scaffold area. It was found that for the PLA membranes (Figure 2A.1), PLA—A membranes (Figure 2A.2), and PLA—B membranes (Figure 2A.3) had 60.2, 51.45, and 44.38% of pure PLA membranes area respectively. Diffractogram fitting was performed in order to quantify the percentage of area formed under the curve where the characteristic PLA peaks are identified. For this, the deconvolution method with Gaussian function was used in a software Origin 8.0, obtaining curves with *R*^2^ = 0.99. The study of this device has been used as a tool in several works of our research group (Silva et al., 2018, 2019a,b).

**Figure 3:**
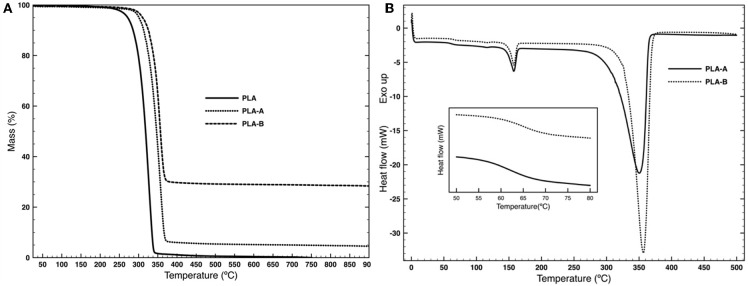
**(A)** TGA curves from 25 to 900°C performed at a heating rate of 10°C min^−1^ under nitrogen atmosphere with a flow rate of 20 mL min^−1^ and **(B)** DSC thermogram from 0 to 500°C and the glass transition region between 40 and 80°C (inset) performed at a heating rate of 10°C min^−1^ under nitrogen atmosphere with a flow rate of 20 mL min^−1^ of the of PLA/TiO_2_ samples.

Table 3 and Figure 3 display the result of the thermal characterization of the sample containing TiO_2_ nanoparticles. The TGA result shows that the degradation temperature of the composite nanofiber (peak around 355°C against the peak at 326.6°C of the pure PLA) is significantly affected by the presence of TiO_2_ but not by its concentrations in the polymer matrix (Laske et al., 2015; Wacharawichanant et al., 2017; AnŽlovar et al., 2018). It is clear from Table 3 that the residual weight expressed in mg/mg is proportional to the TiO_2_ content on the PLA electrospun nanofibers.

**Table 3:** Main DSC thermal transitions (*n* = 3; S.D. < 1%), TGA mass loss temperature peaks and residual weight (R_w_) at 900°C of samples (*n* = 3; ± S.D).

**Sample**	**T_**Onset**_ (°C)**	**T_**Peak**_ (°C)**	**T_**Endset**_ (°C)**	**ΔH** **(J g^**−1**^)**	**T_**g**_ (°C)**	**DTG T_**Max**_ (°C)**	**R_**w**_ 900°C (%)**	**R_**w**_ 900°C (mg/mg)**
PLA	132.8	154.9	170.1	57.7	55.3	326.6 ± 2.6	0	7.2/0
PLA—A (1st peak)	146.6	157.4	163.3	44.8	61.8	354.8 ± 5.6	5.0 ± 0.6	8.95/0.45
PLA—A (2nd peak)	308.1	350.5	364.4	638.8				
PLA—B (1st peak)	148.6	157.6	162.9	35.6	65.8	354.9 ± 2.2	28.2 ± 0.4	8.15/2.30
PLA—B (2nd peak)	330.8	356.8	368.8	540.2				

The DSC results display a glass transition temperature (Tg) and two endothermic peaks in the second heating cycle. The Tg (Figure 3B inset) increase with the amount of TiO_2_ in the PLA matrix from the 55.4°C of pure PLA to the 61.8°C of PLA-A and 65.8°C of PLA-B. The first peak, attributed to the melting point of PLA, is slightly affected by the presence of the nanoparticles (peak around 157.5°C against the peak at 154.9°C of the pure PLA). The second one, attributed to the PLA degradation, has sharper peaks and shifts to a higher temperature in the presence of TiO_2_. The introduction of higher concentration of TiO_2_ in the PLA structure significantly decreases the melting enthalpy of both peaks.

**Figure 4:**
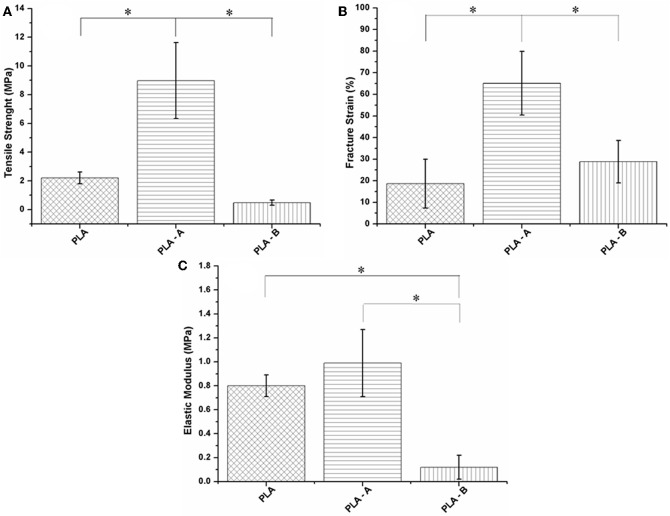
**(A)** Variation in tensile strength (TS), **(B)** fracture strain (FS), and **(C)** elastic modulus (EM) of PLA nanofibers. Statistical test: One-way ANOVA followed by post-test multiple Tukey comparisons *P < 0.05.

The Figure 4 illustrates the mechanical properties (FS, EM, and TS) of PLA/TiO_2_ nanofibers with different TiO_2_ contents. As can be seen, the mechanical properties of the scaffolds were affected by the addition of TiO_2_. There is an increase in the value of these properties for PLA—A and subsequently a reduction for PLA—B. The changes in evaluated values are summarized in Table 4.

**Table 4:** Mechanical properties analysis of PLA—A and PLA—B over neat PLA scaffolds.

**Scaffolds**	**Fracture strain (%)**	**Elastic** **modulus (MPa)**	**Tensile strength (MPa)**
PLA	18.59	0.80	2.20
PLA—A	+250%	+23%	+308%
PLA—B	+55%	−85%	−78%

### Cytotoxicity and Gene Expression Analyses

**Figure 5:**
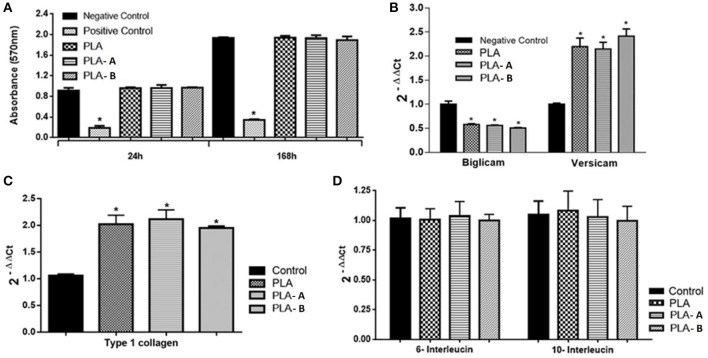
**(A)** Cell viability assay performed by the MTT assay (PLA, PLA—A, and PLA—B). One-way statistical analysis ANOVA and post-test of Tukey Multiple Comparison *P < 0.05 (compared to cells); **(B)** mRNA expression of the versicam and biglicam genes in L929 cells in the control groups and in the PLA, PLA—A, and PLA—B groups. **(C)** mRNA expression of the versicam and biglicam genes in the L929 cells in the control groups and in the PLA, PLA—A, and PLA—B groups. All groups were compared to the control group (cells only). **(D)** Expression of the interleukin-6 and−10 mRNA in L929 cells in the control groups and in the PLA, PLA—A, and PLA—B groups. Statistical test: One-way ANOVA followed by post-test multiple Tukey comparisons *P < 0.05 (compared to control).

Cytotoxicity studies were performed at two different time points (24 and 168 h) and the analyzed groups were compared to a positive control (latex, Figure 5A). The expression of the genes of interest was studied using RT-qPCR from cDNA obtained by the reverse transcription of mRNA obtained from the fibroblast lineage. Before initiating the RT-qPCR reactions, expression of these genes was analyzed by semi-quantitative or end-point RT-PCR to ensure they were expressed (data not shown). The expression of extracellular matrix Versicam, Biglicam type 1 collagen, interleukins-6, and interleukins-10 were analyzed. It was observed that the expression of Versicam increased when L929 fibroblast cells were cultivated on PLA and PLA—A and PLA—B when compared to the control (only cells, *p* < 0.05, Figure 5B). Meanwhile, over expression of type I collagen (COL-1) occurred in the fibroblastic cells in contact when cultivated on PLA and PLA—A and PLA—B (*p* < 0.05, Figure 5C). No statistical differences were observed when the expressions of interleukins-6 and−10 were analyzed (Figure 5D).

### Histological Analysis

The groups were compared qualitatively to check for similar histological aspects. The figures show an overview of the control material and nanocomposites.

**Figure 6:**
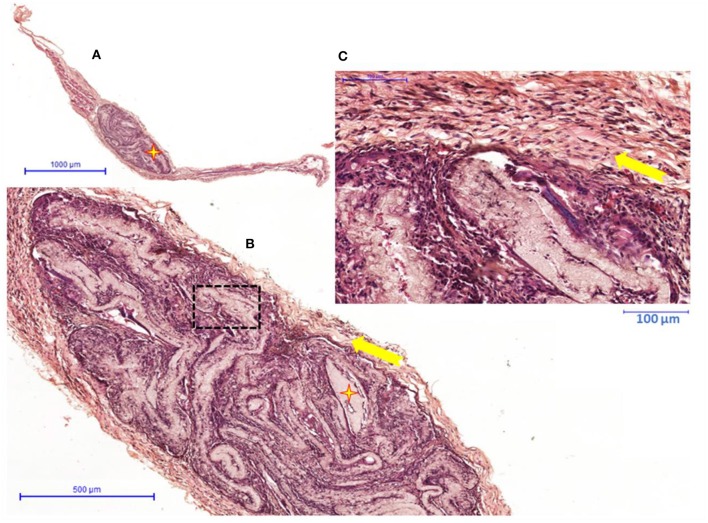
PLA—**(A)** Overview hematoxilin and eosin staining histological image of connective tissue with biomaterial (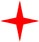) implanted; **(B)** Detail of representative histologic section of connective tissue (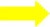) forming the capsule around of biomaterial (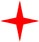); **(C)** Capsule detail with inflammatory infiltrate discrete (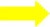).

**Figure 7:**
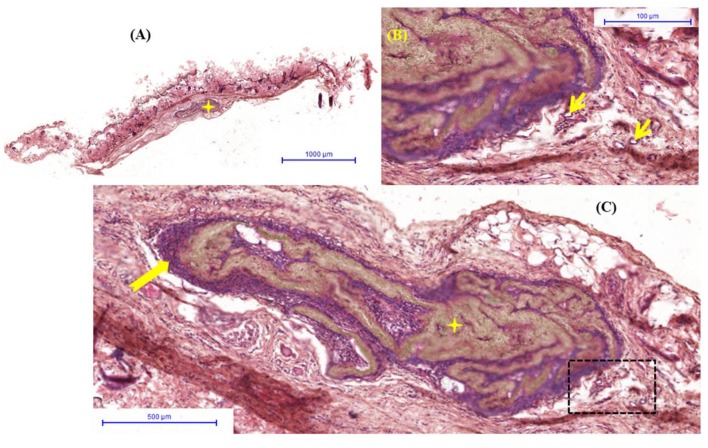
PLA—**(A)** Overview hematoxilin and eosin staining histological image of connective tissue with biomaterial (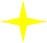) implanted; **(B)** Detail of representative histologic section of connective tissue (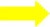) forming the capsule around of biomaterial (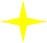) with inflammatory infiltrate discrete; **(C)** Capsule reveals details of neoformed blood vessels (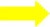).

**Figure 8:**
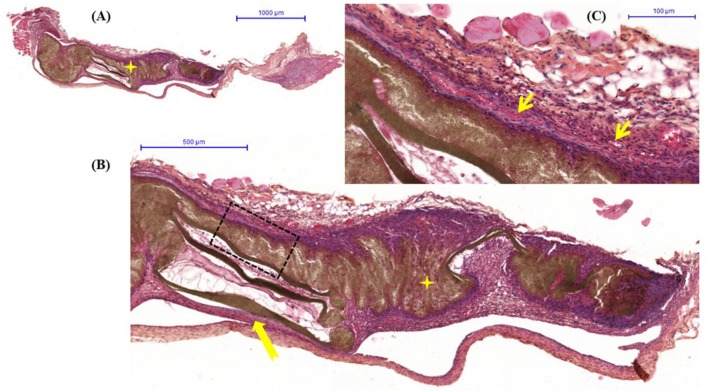
PLA—**(A)** Overview hematoxylin and eosin staining histological image of connective tissue with biomaterial (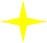) implanted; **(B)** Detail of representative histologic section of connective tissue (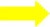) forming the capsule around of biomaterial (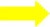) with inflammatory infiltrate discrete; **(C)** Capsule reveals details of neoformed blood vessels (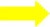).

Clinically, the animals showed no signs of infection, and no foreign body reaction was observed under a microscope (Figures 6–8). A capsule of connective tissue was observed around the membranes of all the three types, indicating a close contact between the material and the surrounding connective tissue. The presence of discrete inflammatory infiltrate was also observed. However, the histological sections of PLA—A (Figure 7) and PLA—B (Figure 8) showed the newly formed vessels, suggesting a higher rate of metabolic activity in this tissue (compared to control, Figure 6). These observed differences are positive events that occurred in the regenerative process, influenced by presence of PLA/TiO_2_ membranes.

## Discussion

None of the electrospun membranes showed beads formation, indicating that the work distances and applied voltage were appropriately chosen (Schuster et al., 2003). It was also observed that the solvents had completely evaporated during the electrospinning process, resulting in fiber diameters with little variation (Schuster et al., 2003; Efron and Moldawer, 2004; Zhang and An, 2007). The decision to keep the membranes for 15 h in a vacuum chamber resulted in the elimination of any residual liquid present in the nanofibers (Efron and Moldawer, 2004; Zhang and An, 2007). The incorporation of TiO_2_ nanoparticles also did not promote bead formation, as seen in Figures 1A–C, indicating that our strategy to disperse these particles using ultrasound resulted in homogeneous dispersion of TiO_2_ nanoparticles inside PLA fibers—as seen in Figures 1B.1,C.1—without inhibiting the PLA behavior—as shown by the XRD images (Figure 1E). A discrete reduction in contact angle was observed while using water when TiO_2_ nanoparticles were incorporated into the PLA (Figure 1D). The XRD deconvolution analysis showed that the addition of TiO_2_ interrupted the arrangement in the PLA polymer backbone by modifying its crystallinity (Baskaran et al., 2006). This intensity was assessed by deconvolutions of the XRD of Figure 2A. Crystallinity plays a very important role in the physical properties of biodegradable polymers—especially the thermal and mechanical behavior—and also affects their biodegradability (D'amico et al., 2016). The addition of 10 and 35% w/w of TiO_2_ on the PLA matrix resulted in a significant decrease of crystallinity index of about 2 and 8%, respectively. Electrospun PLA exhibited two α crystal reflection peaks at 14.0 and 16.8° and a small phase peak at 25.0° due to the high degree of deformation that the electrospinning process causes to the material (Figure 1E). The positive shift of higher values of 2θ and the high degree of crystallinity of the of the electrospun nanofibers of PLA compared to the PLA films can be ascribed to the higher stretching of the polymer chains resulting in higher degree of molecular organization (Oliveira et al., 2013; Farid et al., 2018).

Thermogravimetric analysis (Figure 3A) showed that the degradation of PLA containing TiO_2_ nanoparticles takes place in a well-defined single step with a derivative thermogravimetric (DTG) temperature peak at around 355°C (Table 3) with significant differences between nanoparticles content and pure PLA that showed a lower degradation peak at around 327°C in accordance with the DSC results (Mofokeng and Luyt, 2015; Zhang et al., 2015). After degradation, the weight remained constant until 900°C and leading to a residue content in function of the TiO_2_ concentration (Table 3). PLA lead to a 0% residue at this temperature as previously observed (Virovska et al., 2014). With an increase of TiO_2_ on the PLA nanofibers, there was an increase in residue produced which supports the presence of the nanoparticles in the nanospun fibers structure (Costa et al., 2013).

During the cooling cycle in the DSC analysis, no crystalline structures or other transitions appeared (data not shown). The DSC thermogram during the second heating shows two endothermic peaks at 157 and 350°C, indicating the melting and degradation peaks of PLA, respectively (Gupta et al., 2007). The inclusion of TiO_2_ showed small differences in the melting peak (157°C) compared to the pure PLA (155°C). However, comparing the 10 and 35% TiO_2_ containing PLA nanofibers, the degradation peak displayed a slight increase (from 351 to 357°C) and a large positive shift of around 20°C in the onset temperature (from 308 to 331°C). The Tg of PLA nanofibers showed a significant increase with the addition of TiO_2_ in the polymer matrix (Table 3). This suggests an interaction between TiO_2_ and PLA matrix (Zdraveva et al., 2018; Kaseem et al., 2019). These interactions restrict the mobility of the molecular chains in the PLA amorphous segments enhancing the cooperative motions of the chains which require much more activation energy to occur (Gasmi et al., 2019).

Moreover, the significant increase in decomposition onset temperature and the decrease in both enthalpy and crystallinity of the PLA composite with higher TiO_2_ nanoparticles reinforce the hypothesis that there was an efficient inclusion of intermolecular bonding with the PLA matrix due to the anti-plasticizing effect of TiO_2_ nanoparticles (El-Sayed et al., 2011; Amin et al., 2019).

Several researches have studied how addition of nanoparticles can improve mechanical properties in ultra-thin polymeric fibers. It has been proven that, the improvement in mechanical properties of PLA—A over neat PLA in this study was attributed to the favorable interactions between the polymer matrix and the homogeneous distribution of TiO_2_ nanoparticles (as augmented in the internal friction) within the fibers as a filler, showed in the Figure 1C.1, making it toughest and most flexible (Ramier et al., 2014; Sadeghi and Shahedi, 2016; Feng et al., 2019). The reduction of TS, FS, and EM in PLA—B (Figure 4 and Table 4) can be attributed to an anti-plasticizing effect, in which nano-TiO_2_ might play the part of an anti-plasticizer due to increased interaction, a decreased the free volume between chains, a reduction in film flexibility and reduction in crystallinity, showed in Figure 1E, making it less tough (Shaili et al., 2015; Feng et al., 2019).

The membranes did not cause any decrease in the number of cells when compared to the control group in the cytotoxicity assay (Figure 5A). Cytotoxicity or evaluation of toxicity in cell culture is a complex *in-vivo* phenomenon that manifests a broad spectrum of effects, from cell death to metabolic aberrations—i.e., no cell death but functional changes (Kao et al., 2007). All groups of materials (PLA—A, and PLA—B) caused an over expression of the versicam mRNA in fibroblasts when compared to the control group. On the other hand, the biglicam showed a decrease in expression in the fibroblasts (down expression) when in contact with the studied nanocomposites in all three groups (Figure 5B). Type-I collagen was upregulated in all the membranes (Figure 5C). The electrospun membranes, however, did not show differences from control when analyzed for 6- and 10-interleukins (Figure 5D).

Type-I collagen plays an important role in maintaining the integrity of the extracellular matrix. Type-I collagen has a fibrillar type structure and is the most investigated type of collagen due to its abundance and the fact that it is the main structural element of several tissues. It is expressed in almost all connective tissues and plays a key role in the skin repair processes (Wong et al., 2013). Versicam is present in the dermis (Ruoslahti, 1989) and has important biological functions in the regulation of skin behavior (Bianco et al., 1990; Kinsella et al., 2004). Recent studies have shown that versicam interacts with leukocytes, promoting their adhesion. In addition, the incorporation of versicam into the ECM blocks monocyte adhesion and attenuates the inflammatory response. When binding to hyaluronic acid, versicam influences the T lymphocytes, aiding these cells to synthesize and secrete cytokines that assist the immune response. Versicam is emerging as a potential target in the treatment of inflammation, promising broad therapeutic benefits in the future due to the fact that it is an ECM molecule that plays a central role in the inflammatory process (Wight et al., 2014). The upregulation of versicam and type-1 collagen can be attributed to the activation of connective tissue formation, presumed to be related to repair of wounds and fibrotic diseases of the skin (Wahab et al., 1996).

A study conducted in 2001 compared down regulation of the decorin gene mRNA expression in post-surgical regenerated fibroblast cells in comparison to healthy human gingiva. The expression of mRNA for the versicam presented increased expression (upregulation) in this study (Ivanovski et al., 2001). In this study, the expression of versicam and biglicam genes corroborate with observations by Ivanovski. In our study, it was observed that all of the three groups of membranes (PLA, PLA—A, and PLA—B) caused over expression of the versicam in the fibroblasts when compared to the control group (Figure 5B). These findings are also in agreement with previous studies where the downregulation of decorin mRNA and upregulation of versicam in gingival cells and periodontal ligament cells were also observed (Haase et al., 1998). Other studies also report the correlation between exposure of growth factors, rates of cell proliferation, and synthesis of proteoglycans in other cell lines (Kähäri et al., 1991; Mauviel et al., 1995).

The gene expression findings of interleukins in the present study demonstrate that there is no change between the control and nanomaterials groups. The cited references support the idea that the developed membranes do not cause an inflammatory response in the cells of the fibroblast line used. This is an important property of a material that can be used in dressings in the future, since it would avoid problems related to scarring—such as excessive inflammatory response—that would delay the regenerative process (Kopf et al., 2010; Scheller et al., 2011).

TiO_2_ has been proven to be a nanoparticle that can to modulate the immune functions, it is dependent to concentration, dose or route of administration (Lappas, 2015). The ability of TiO_2_ nanoparticles in prove reactive oxygen species (ROS) and increase membrane permeability maximize antibacterial activity and improve the wound healing as was observed previously (Sankar et al., 2014). Moreover, the TiO_2_ nanoparticles could cause enhanced blood coagulation, which is an important first step in the wound healing process (Seisenbaeva et al., 2017). Our *in vivo* results were (Figures 4–6) similar to Seisenbaeva et al. (2017) and in the study, it was observed that TiO_2_ improved wound healing. We confirmed that the electrospun membranes with TiO_2_ can stimulate and modulate inflammation, which is very important for human health, since there is bigger formation of blood vessels (Babelova et al., 2009; Moreth et al., 2014).

Various biological and synthetic skin replacements are available commercially available. Although there are over 3,000 types of dressings on the market, there is no product that is effective for treatment of chronic wounds such as venous leg ulcers, diabetic wounds, and pressure ulcers and burns. The membranes discussed in this study are ideal candidates for curative materials in the care of difficult-to-treat wounds and aiding the healing process and helping patients and health care professionals (Dhivya et al., 2015).

## Summary and Conclusions

Three different membranes types were evaluated: One with PLA nanofibers but without TiO_2_ content, and two with PLA and varying concentrations of TiO_2_ (10% and 35%—w/w). A higher concentration of TiO_2_ in the PLA structure significantly decreases the melting enthalpy of PLA. PLA with 10% of TiO_2_ improved in more than 300% the tensile strain compared to PLA. All three membranes were found to be non-toxic against fibroblast L929 cells. The membranes also increased mRNA expression in Versicam and type-1 collagen, which are both important for the tissue repair process. It was also observed that the membranes did cause inflammations as demonstrated by the absence of alterations in the expression of interleukins-6 and−10. *In vivo* analysis indicated that our membranes can be used as materials for wound healing applications, as there were no inflammations observed, and the formation of blood vessels was identified.

## Data Availability Statement

The datasets generated for this study are available on request to the corresponding author.

## Ethics Statement

The animal study was reviewed and approved by 10/2015-CEUA/ICT/CJSC-UNESP.

## Author Contributions

All authors contributed to the design of the study, writing of the manuscript, and read and approved the final manuscript. TM and RR performed the biological *in vitro* and *in vivo* tests. TT, CE, AS, AF, AZ, and LM produced and characterized all membranes. LV, ES-F, FM, TW, and AL supervised all students.

### Conflict of Interest

The authors declare that the research was conducted in the absence of any commercial or financial relationships that could be construed as a potential conflict of interest.
